# Implementing QR codes in academia to improve sample tracking, data accessibility, and traceability in multicampus interdisciplinary collaborations

**DOI:** 10.1371/journal.pone.0282783

**Published:** 2023-04-06

**Authors:** Cristian Hernandez, Elham Aslankoohi, Pavel Frolikov, Houpu Li, Sri Kurniawan, Marco Rolandi

**Affiliations:** 1 Department of Electrical and Computer Engineering, Baskin School of Engineering, University of California, Santa Cruz, Santa Cruz, California, United States of America; 2 Department of Computational Media, Baskin School of Engineering, University of California, Santa Cruz, Santa Cruz, California, United States of America; Jazan University Faculty of Computer Science, SAUDI ARABIA

## Abstract

The growing number of multicampus interdisciplinary projects in academic institutions expedites a necessity for tracking systems that provide instantly accessible data associated with devices, samples, and experimental results to all collaborators involved. This need has become particularly salient with the COVID pandemic when consequent travel restrictions have hampered in person meetings and laboratory visits. Minimizing post-pandemic travel can also help reduce carbon footprint of research activities. Here we developed a Quick Response (QR) code tracking system that integrates project management tools for seamless communication and tracking of materials and devices between multicampus collaborators: one school of medicine, two engineering laboratories, three manufacturing cleanroom sites, and three research laboratories. Here we aimed to use this system to track the design, fabrication, and quality control of bioelectronic devices, *in vitro* experimental results, and *in vivo* testing. Incorporating the tracking system into our project helped our multicampus teams accomplish milestones on a tight timeline via improved data traceability, manufacturing efficiency, and shared experimental results. This tracking system is particularly useful to track device issues and ensure engineering device consistency when working with expensive biological samples *in vitro* and animals *in vivo* to reduce waste of biological and animal resources associated with device failure.

## Introduction

Modern research encourages multicampus collaboration amongst different disciplines to share concepts, methods, and answer more complex questions that cannot be answered within a single discipline [1]. If multicampus interdisciplinary research is not executed properly the produced results and milestone completion success rate can be jeopardized due to inadequate project coordination. One of the biggest challenges of project coordination is communication between experts across different fields that often do not share terminology and tools. Multicampus communication is critical when devices and samples are transferred between different laboratories. For example, it is necessary for researchers that require a device for in vivo testing to receive the proper protocols and understanding of the device’s functionality from engineers that fabricated and tested the device in a different laboratory. This exchange happens often in bioengineering disciplines in which materials and devices are typically developed in an engineering laboratory and are used in a biological or medical setting [2] such as in tissue engineering [3, 4], medical devices and sensors [5] and bioelectronics [6–8]. Typically, this exchange of information happens in person via exchange of personnel between laboratories so that collaborators can understand and trust each other through socialization [9]. However, the recent COVID pandemic has not only hampered travel and exchanges across different laboratories and campuses, but has forced most researchers in the same laboratory to work alone due to limited room capacity restrictions. This limited interaction has made successful exchange of information, materials, and samples more challenging with high risk of failed experiments due to incomplete communication and lack of device traceability between collaborators. This limited interaction, data traceability, and data accessibility is adding pressure to academic researcher to deliver on multidisciplinary projects that are being funded by governmental institutions [9]. The larger in scale the project is the more important accurate research data management becomes to the funding agencies [10]. Research data management is the concept that aims to create an accessible, documented, organized research environment with reusable quality research data. Funding institutions demand open access to research results and data in order to see if the project is yielding results that are still worth funding [10]. Academic researchers find it challenging to balance performing scientific work and managing the results. Sharing data at various stages of experimentation and explaining to colleagues from different institutions is difficult to do, especially if it is with individuals that they have never worked with before.

To mitigate some of these challenges, project management with a tracking system for devices and data can allow interdisciplinary teams to perform quality work while reaching task milestones on time and staying within budget [11, 12]. Additionally, project management tracking can reduce the need for excessive post pandemic travel and reduce carbon footprint of research activities. For example, QR codes have become a common tool to share information and are easily accessible with any modern smartphone [13]. In academic settings, QR codes provide data on classroom links, tutorials, vCard contacts, uniform resource identifiers, e-mail addresses, map directions, text, and chemicals. Academic libraries use QR codes to help students access their services [14–19]. QR codes are also being used to track student attendance and increase student collaboration and engagement [20, 21]. Students have successfully incorporated QR codes into their projects [22–26]. Examples include: a handheld augmented reality supported English language learning system with the use of QR codes [27], a periodic table of elements that contained a QR code for every chemical [28], QR codes implemented into a mobile phone application for classroom management, communication [29], A formative class assessment using questionnaires and shuttle card called “Daifuku-cho” with mobile phones and QR codes in Japan [30].

QR codes have started to surface in the research environment for quick tracking of biological specimens [12, 31]. However, current use of QR codes, project coordination tools, and research data management in academic research remains limited. The limitation on the ability of data transfer between collaborators hampers the project’s success. If a mistake or failure is documented but not shared due to lack of accessibility or communication to others then the knowledge to mitigate those failures is not being used to learn how to avoid them [32]. Essentially, easily accessed data stored within QR codes can benefit a research labs production by providing best practices for fabrication and testing on their smart phones [33]. Here, we introduce a QR code tracking system for materials and devices as a solution to improve project coordination across five collaborating universities: UC Santa Cruz, University of Montana, University of Utah, Tufts University, and UC Davis during the COVID pandemic as part of a DARPA multicampus project associated with the BETR program. This project involves the use of bioelectronic sensors and devices [34, 35] combined with AI algorithms to speed up wound healing [36]. Bioelectronic devices are designed at UC Santa Cruz; fabricated with up to ten clean room processes at University of Montana, University of Utah, and UC Santa Cruz; tested at UC Santa Cruz; and then shipped either to Tufts University for *in vitro* experiments or to UC Davis for *in vivo* work. Inspired by Toyota’s Total Production System [37], we introduced device QR codes as a device “Kanban” to solve two main issues: (1) inventory management to confirm devices are ready “just in time” when the biological experiments are scheduled to avoid poor performance due to device age or missed experiments due to delays in fabrication, and (2) the need to trace the root cause of device failure during biological experiments (e.g., device leakage, malfunctioning electronics) to specific steps in device design or fabrication.

This system focuses on traceability of design, fabrication, quality control, and shipping which are often neglected in academia. Traceability is neglected because research universities adaptive capacity is threatened by an ingrained social behavior that lacks desire for innovation in the organization and practices at universities [38]. If a system doesn’t fall within the status quo of an academic research laboratory then it can be difficult for the principal investigators and graduate students to buy into using the system. The QR code tracking system succeeded at UCSC because it solved the problem that researchers faced when starting a multidisciplinary project. The challenge was how does a research team keep track of all device fabrication, quality control, test data and then make all this data accessible and traceable in a manner that can be easily understood by other teams. The result is the use of two project management tools, QR Code Generator and Asana, that then led to our UCSC application that allows both tools to integrate data input into the QR Code Generator. Asana is a platform that provides work teams an online space to organize, track, and manage project tasks. The contribution our research provides to academia is feedback on mitigating issues that arise from multidisciplinary projects through tracking system development that targets traceability of design, fabrication, quality control, and shipping via integration of QR codes and Asana project management tool.

## Materials and methods

### QR code generator

We generate the QR codes that help track data for our multicampus teams via an outsourced management tool desktop app, https://www.qr-code-generator.com/. The project management tool provides dynamic functionalities allowing the codes to be edited even after having been printed onto a label. This capability is useful for devices that go through various fabrication steps and quality control checkpoints before completion. It allows the fabricator to make updates throughout the products lifecycle without having to continuously reprint the QR Label. The QR code generator allows the user to customize the information: QR code name, basic information, categories, and contact information displayed on the scanning phone. We use a unique identifier known as a batch number to name the QR code. Batch numbers keep track of devices that are built in bulk. This study requires assigning batch numbers to various wafers fabricated at the same time. A wafer yields seven devices once completed. The batch number consists of nomenclature that provides relevant information on the wafer’s fabrication. The batch number’s nomenclature, 123456-7-8-ABC-9, represents the following:

1. 123456: The digits provide the date that manufacturing started on the wafer.
2. 7–8: The digits identify the number of wafers that started fabrication on the same date.
3. ABC: The letters are abbreviations that provide the device’s design name.
4. 9: The final digit conveys the device’s design version.

At a certain step of the fabrication process, wafers are diced into individual devices and each device is assigned a part number. The part number’s nomenclature is the batch number but with an added letter at the end ranging from A-G to represent the seven devices, for example123456-7-8-ABC-9-D. Without these two unique identifiers the devices would not be traceable with the QR code tracking system. The user interface provides the following customized categories when scanning the label: (1) batch information, (2) wafer build process, (3) device build process, (4) batch tracking, (5) quality control, (6) shipping schedule, and (7) sample testing. A label maker prints the QR code and batch number. A one-inch label goes on a plastic container that protects the wafer throughout its lifecycle. The wafer goes through fabrication, quality control and is diced into individual devices. The fabricators assign each device with a new unique identifier that is known as a part number. The individual devices continue fabrication and quality control until completion and get shipped to the collaborators for *in vitro* testing. During a device’s production lifecycle, all the categories are updated manually in the QR code generating desktop app and Asana. Asana is an outsourced online application that helps keep projects on track via task assignments, deadlines, and data sharing [39, 40].

### Desktop and mobile

We developed at UC Santa Cruz an application that integrates data from QR codes onto Asana. Our application mirrors functions from the outsourced QR code generator, but allows the user to make updates on a mobile phone and automatically transfers the data onto Asana. The UCSC application is opensource and will provide users an opportunity to integrate it into their projects for free after the finalized version is complete. Our goal is to also introduce the outsourced QR code generator that this study first started using to track device data to provide other academics with options to help manage their projects. The UCSC integration application is further explained in the results section.

## Results and discussion

**Fig 1:**
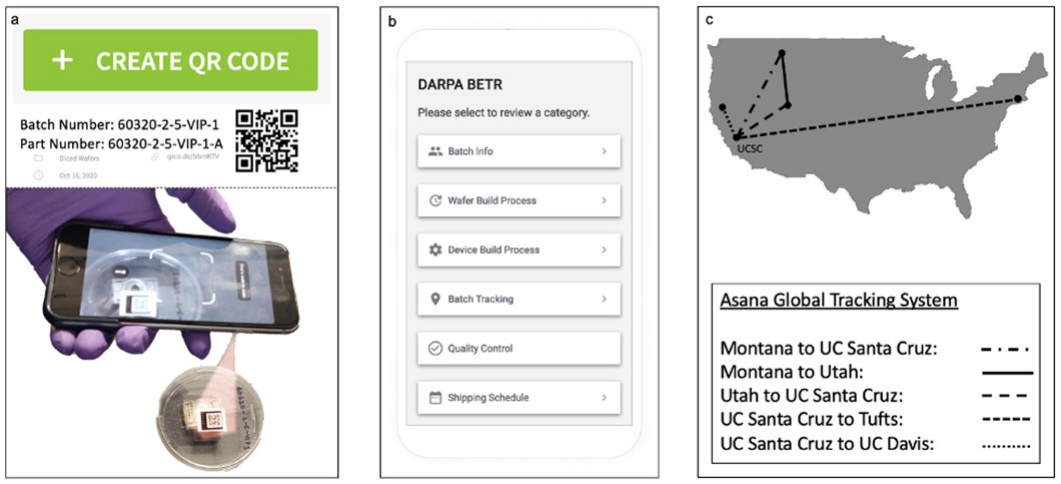
Tracking system overview. (a) Generate QR Code on subscription-based desktop application and label device container with unique identifier and QR Code. Scan QR Code to access data. (b) Data accessed focuses on the following categories: batch info, build process, batch tracking, quality control, shipping schedule, and sample testing. (c) Data from the QR codes tracking system is implemented into Asana, a global tracking system that we customized to provide device data traceability amongst five universities. Asana tracks the location and transit of the device across the different collaborating institutions. This tracking is important for inventory management to make sure that there are always enough devices at each step and location so that the subsequent fabrication step or experiment is not delayed. Fig 1b provided through courtesy of QR Code Generator.

We used outsourced project management tools, QR code generator and Asana, to track all bioelectronic device production lifecycles to ensure engineering device consistency to reduce waste associated with device failure (Fig 1). The QR generator generates a QR code and assigns a batch number for any wafer and tracks the wafer throughout its lifecycle. The researcher can scan the QR label at any time during the products lifecycle to access any information needed to make a decision (Fig 1a). The QR code consists of different parts that correspond to different steps of the process such as batch info, wafer build process, device build process, batch tracking, quality control, shipping schedule, and sample testing (Fig 1b).

**Fig 2:**
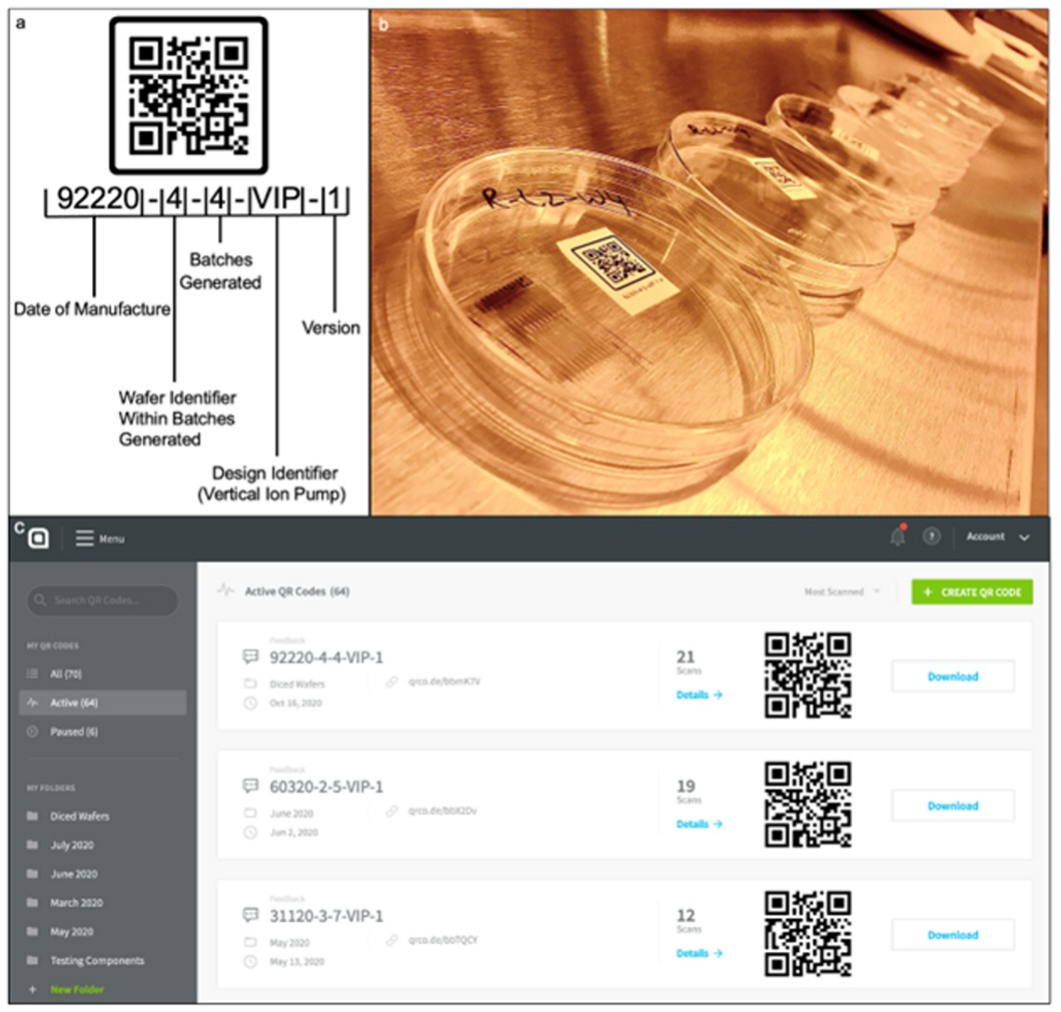
QR code tracking system. (a) A detailed breakdown of the unique identifier’s nomenclature that is tied to a QR Code, and (b) label device container with unique identifier and QR Code. (c) User interface subscription-based desktop application used to generate QR Code. Fig 2C provided through courtesy of QR Code Generator.

In the first step of the tracking process, the researcher that fabricates a device also prints a QR code with a unique identifier (Fig 2a). The batch number identifies and traces the bioelectronic device’s production history and relevant issues that occurred during manufacturing and testing. The nomenclature provides instant information on the wafers to determine the following: date of manufacture, wafer identifier, number of wafers in production, design identifier, and design version. For example, 92220-4-4-VIP-1 allows our fabricators to determine wafer fabrication started on September 22, 2020, it’s the fourth wafer out of four batches produced, and it’s the first design version of the Vertical Ion Pump, which is a bioelectronic device design. The devices are tracked by placing a label on the plastic containers that secure and protect the devices throughout production (Fig 2b). The outsourced project management tool allows the researchers to generate and update a QR code (Fig 2c). QR codes are generated by selecting the button titled “Create QR Code.” This user-interface then provides a customizable layout that can be edited and reedited with data pertaining to a team’s needs.

**Fig 3:**
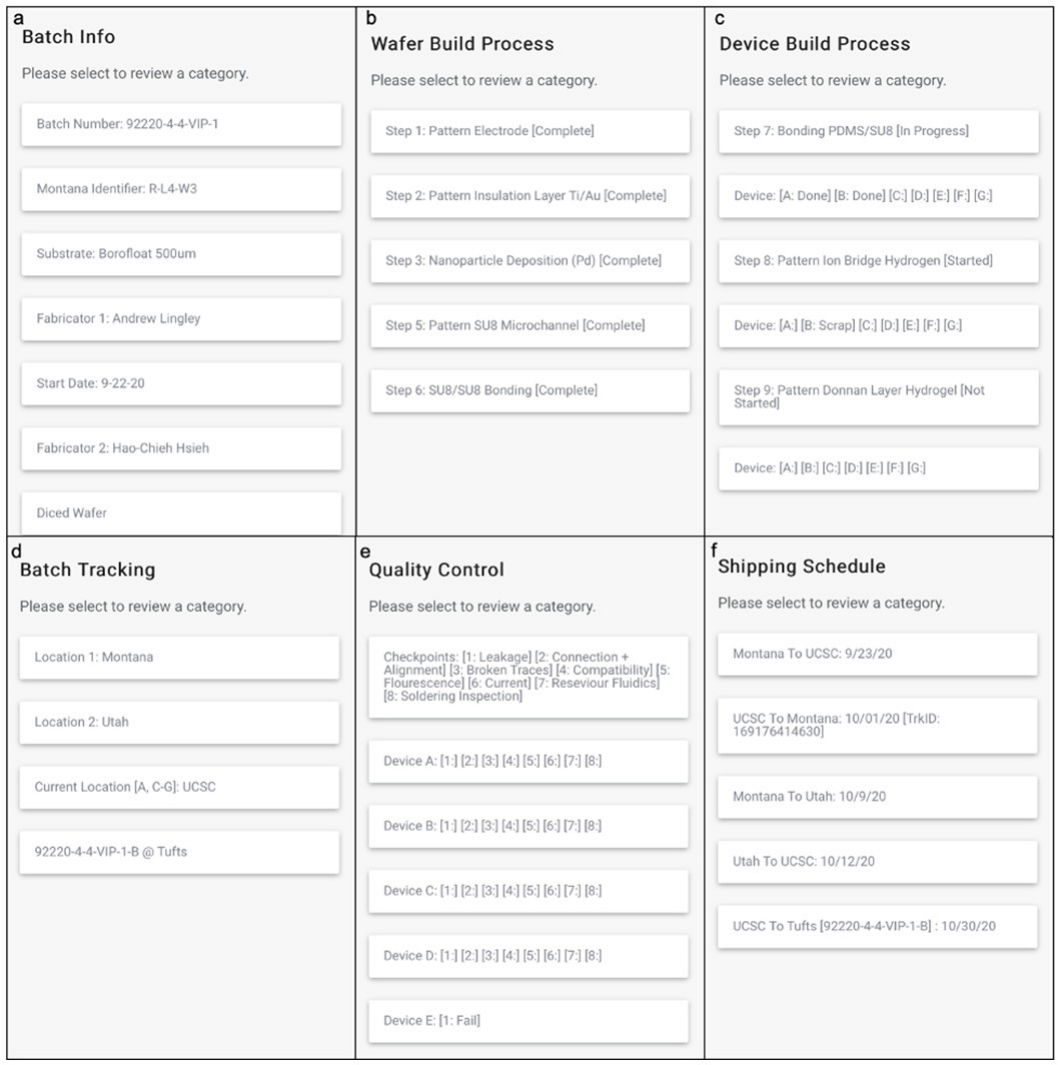
Categoric data points that QR code system tracks. (a) Batch Information, (b) Wafer Build Process, (c) Device Build Process, (d) Batch Tracking, (e) Quality Control, & (f) Shipping Schedule. Fig 3 provided through courtesy of QR Code Generator.

The QR code system tracks data that has been separated into categories to keep the information organized (Fig 3). Batch info provides the researcher with general data such as batch number, initial fabrication location, substrate material, fabricator name, etc. (Fig 3A). For example, Fig 3A lets the researcher know that wafer 92220-4-4-VIP-1 was initially fabricated in the Montana cleanroom on September 22,2020. The wafer substrate is Borofloat 500 μm. The device has been shipped to Utah to continue more fabrication and was diced into seven devices that received part numbers. The wafer build process consists of six steps and the system displays the wafer’s progress through the fabrication process (Fig 3B). Completing the first six steps leads to the wafer being diced into seven devices that receive new part numbers. The system tracks the fabrication process of each diced device and if any failures occur during a specific step (Fig 3C). The wafer and the devices it yield after dicing are tracked throughout fabrication and testing to determine which collaborators have come in contact with the device(s) (Fig 3D). The system tracks quality control performed on the devices to determine the cause of failure and at which step during production the device(s) failed (Fig 3E). Shipping for the device(s) is tracked to help researchers plan for fabrication or testing based on the package’s arrival (Fig 3F). As the project continues more categories will be added. This tracking is important so that if any issues occur during *in vitro* testing, the researchers are able to track down all of the specific processes and dates that that wafer has undergone. For example, if there were any issues with bonding two of the wafers together that would result in leakage of fluid from the device, the QR code would allow the UC Santa Cruz team to trace the wafer back to a specific device process in the cleanroom in Montana. If leakage were to occur in most of the devices produced with the same process, then we will determine that we required to optimize the process so that the bonding between the wafers is more robust.

**Fig 4:**
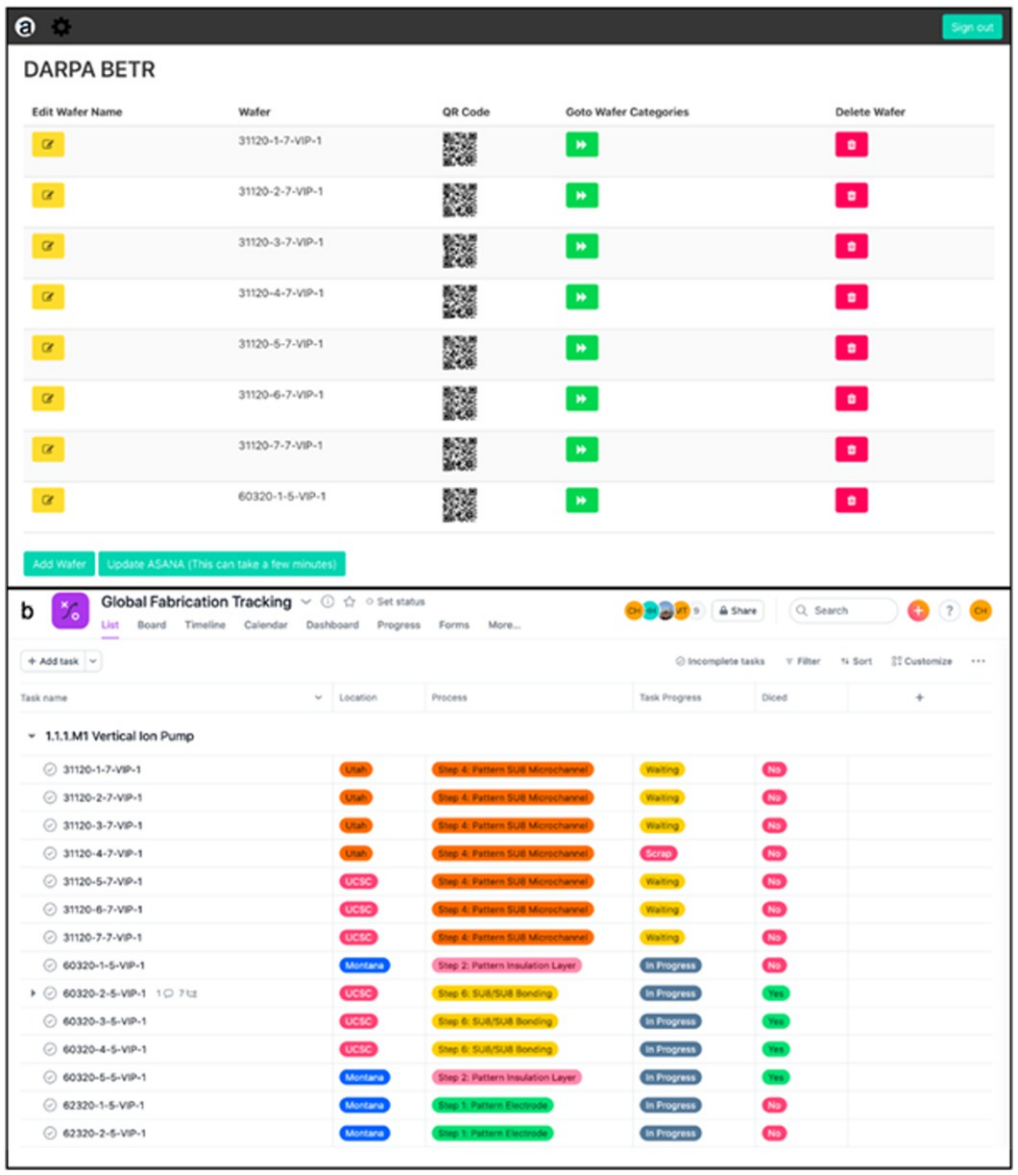
Application Overview (a) Application Tracking System that Integrates QR Codes and Asana, and (b) Asana Global Tracking System. Fig 4b provided through courtesy of Asana.

Using the QR code generating tool, a user can scan the QR code and access the data via mobile phone, the data can only be updated on a desktop website application. All the information then had to be manually inputted into Asana for all the collaborators to easily access and trace data in one organized location. To provide more real time updates, prevent any loss of data, simplify data implementation, and automatically transfer data from the QR code tracking system to Asana, we developed a mobile integration application (Fig 4). The mobile integration application is a web-based user interface that was developed using python -py4web web framework and Vue.JS- JavaScript framework, essentially a simple database with a user-friendly user interface that does not require users to know query-based language to inquire about any categories or data under certain categories. In the early iteration of the system, the integration application only had preset categories. However, upon feedback from a subset of stakeholders, the ability to create any category was added, allowing for a less rigid use-case. This integration application is open-source and will be made available to all interested universities to help academic researchers manage their data better once the final version has been completed. Fig 4 displays the user interface of the application that was made at UCSC. With our application, a user can make updates on the mobile and desktop application. The integration application uses py4web (a database driven web framework) for the backend framework. The QR Code tracker website is a user navigable database so py4web is a perfect fit as the framework.

The user interface for the tracker website was specifically designed to mimic the outsourced QR code generator system with added accessibility. A user can create their own categories and fill them with any data that mitigates project issues directly on a mobile phone. The tracker website uses very little computational power because it uses pythonanywhere.com for hosting. The biggest challenge with our application development came from integrating the Asana API. Our application provides a functionality to automatically transfer all the data on the QR code tracking system into Asana by selecting the “Update Asana” button.

Fig 4b displays a snippet of the data that gets automatically transferred into the project management tool Asana when using the integration application. All the data that is stored and shared between the researchers who focused on fabrication and testing gets added onto Asana. This allows the data to be easily understood and provides easier access to all users across three universities. Ten fields that are filled with data points that allow the users to coordinate the project accordingly. The fields are location, assignee, due date, process, task progress, diced, expected devices, devices yielded, scrap percentage, and testing. Location conveys which university the device(s) are at. Assignee provides the name of the researcher that is in charge of the device(s). Due date is the date the device(s) must finish in order to reach milestones in a timely manner. Process provides the fabrication step the device(s) are currently going through. Task progress details if the device(s) are in progress, not started, done, waiting, or scrap. Diced details if the wafer has been cut into seven individual devices. Expected devices provides the number of devices the wafer needs to generate when finished with fabrication. Devices yielded provides the number of devices that were generated after fabrication completion. Scrap percentage is a basic formula that provides the percentage of failure from a wafer. The formula is ([scrap device(s) / expected devices] * 100). The goal is to keep device failure below 25%, which is the standard industry follows. Knowing the scrap percentage opens up the dialogue on what caused the failure and how can it be prevented. This feature is provided to understand the causes of failure in the first prototype and the hope is that the knowledge will mitigate any risks from the same failure occurring in future fabrications. Testing details if the device will undergo in vitro or in vivo testing.

Before the integration application was distributed widely within our team, the application’s code was tested to ensure that the database and the queries were reliable. After debugging and code checking, the application was tested by two co-authors of this paper. The testing involves investigating the intuitiveness of the icons seen in Fig 4 as well as a walkthrough of the different functions of the application.

**Fig 5:**
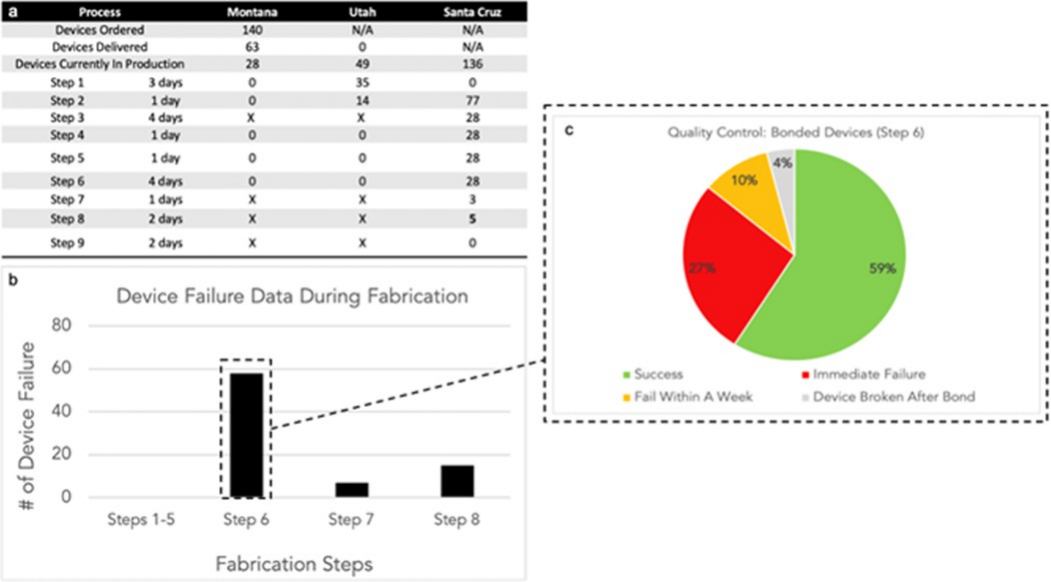
Data obtained from QR code tracking system. (a) Table displaying total quantity of devices in production and current fabrication steps, (b) Graph showing the number of device failure during fabrication process, (c) Pie chart conveying quality control statistics on step 6.

In order to track inventory at different steps, we curate the data from Asana into a table that is shared with the principal investigators of the project (Fig 5a). This table allows all collaborators to know that the Montana cleanroom has delivered 63 devices out of 140 that were ordered. The Montana team plans to start fabrication on 28 devices. The Utah cleanroom has 49 devices in production with 35 on step 1 and 14 on step 2. UCSC cleanroom has multiple devices in production at various steps. This table allows for the researchers that perform device fabrication to organize their fabrication runs to supply the demand for devices for *in vitro* and *in vivo* testing. In turn, it allows for the researchers performing *in vitro* and *in vivo* testing to plan and prepare their experiments accordingly to when the devices will be available.

The QR code tracking system has contributed to our users in the following manner:

1. Accessibility: Data is easily accessible to the user, allowing the user to determine which step of the process the device is in and how to proceed.
2. Traceability: Our users can trace the device process and determine when, where, and produced by whom.
3. Scrap Tracking: User keeps track of failed devices including which step it failed and describes reasoning for failure.
4. Device Selection: Our users can determine which outsourced devices to continue fabrication or testing based on quality control data.

The QR code tracking system has helped to determine step 6 as the most crucial step that our devices can fail (Fig 5b and 5c). It is the fabrication step where the SU8 layer delaminates from another SU8 layer after a PDMS structure has been bonded to it. This graph was only made possible with the ability to easily access and trace the data found in QR code tracking system via Asana. In order to reduce this problem two new quality control checkpoints were incorporated. The first is to view the devices under a microscope and capture a stitched image. The image is then inspected for delamination or cracking. The image is linked to the QR code so the fabricator can view the image and determine whether to proceed with fabrication based on the image and the approval from the individual performing quality control. The second quality control checkpoint is to apply tape to one device out of an entire batch and if the SU8 delaminates then the entire wafer is not used and the manufacturer is contacted to determine if the fabrication protocol was followed. The QR code tracking system has allowed our team to determine that step 6 only has had a 59% bonding success rate, 27% bonding fails immediately, 10% fail within a week, and 4% bond but the devices is broken due to human error. Having this data has pinpointed the root cause of the bioelectronic device failure mode and has improved our team’s focus on how to troubleshoot the issue and communication during meetings. Deciding not to move forward with a device saves time and cost during production and experimentation.

## Conclusion

Multicampus interdisciplinary projects need a QR code tracking system and can stem to benefit in project coordination especially during Covid-19. Here, we describe a QR code tracking system that we used to track device design, fabrication, testing, and *in vitro* and *in vivo* use across five campuses as part of a DARPA funded multidisciplinary project. This system allows for more efficient “just in time” inventory management and faster identification of root cause of failure by tracking the history of a specific device. We believe that this system can help these larger projects achieving a more efficient use of resources by limiting the number of failures. This is particularly important for bioengineering projects that involve engineering devices in biological and animal use. Finally, we hope that improved efficiency may increase the yield of results from federally funded research. A disadvantage for using our QR code tracking system is that with any new system it takes time to get individuals accustomed to incorporating it into their workflow. In industry project management tools are integrated in the workflow and expected, but academic researchers require more time to be convinced on the benefits of integrating QR codes and project management tools in their projects. For this reason, the application interface was developed to be user friendly. This research study was provided to inform how our system and integration application allowed for more efficient decision making due to the statistical analysis of device failure during specific fabrication steps. The statistical analysis pinpointed the root cause of the bioelectronic device failure mode stemmed from step 6 with a staggering 59% failure rate. This shifted our team’s focus on how to troubleshoot the issue and led to the development of a new device version that has been successful for in vivo testing with results that secured our funding for the next phase. We will continue improving the application to its fullest potential.

## Supporting information

S1 File QR code integration application uploaded as a ZIP file. All files in folder are required to run application successfully.
(7Z) Click here for additional data file.
